# Integrating Lifestyle, Mechanistic Therapeutics, and Computational Approaches in Cancer: Highlights from the Irish Association for Cancer Research Annual Conference 2025

**DOI:** 10.3390/cancers18132045

**Published:** 2026-06-24

**Authors:** Cathy E. Richards, Amira F. Mahdi, Neil T. Conlon, Maria Prencipe, Sudipto Das, Marie McIlroy, Jacintha O’Sullivan, Simone Marcone

**Affiliations:** 1 School of Dentistry, RCSI University of Medicine and Health Sciences, D18 NY72 Dublin, Ireland; catherinerichards@rcsi.ie; 2 Limerick Digital Cancer Research Centre, Health Research Institute, School of Medicine, University of Limerick, V94 T9PX Limerick, Ireland; amira.mahdi@ul.ie; 3 School of Biotechnology, Life Sciences Institute, Dublin City University, D09 NR58 Dublin, Ireland; neil.conlon@dcu.ie; 4 School of Biomolecular & Biomedical Science, University College Dublin, Belfield, D04 V1W8 Dublin, Ireland; maria.prencipe@ucd.ie; 5 School of Pharmacy and Biomolecular Sciences, RCSI University of Medicine and Health Sciences, D02 YN77 Dublin, Ireland; sudiptodas@rcsi.ie; 6 Department of Surgery, RCSI University of Medicine and Health Sciences, D02 YN77 Dublin, Ireland; mmcilroy@rcsi.ie; 7 Trinity St. James’s Cancer Institute, Trinity Translational Medicine Institute, Trinity College Dublin, D08 W9RT Dublin, Ireland; osullij4@tcd.ie; 8 School of Biology and Environmental Science, University College Dublin, D04 N2E5 Dublin, Ireland

**Keywords:** cancer research, drug delivery, tumour microenvironment, nanotherapeutics, exercise oncology, cancer stem cells, tumour plasticity, artificial intelligence

## Abstract

The Irish Association for Cancer Research (IACR) Annual Conference 2025, held in Belfast, showcased cutting-edge developments across the cancer research landscape. This report summarizes key presentations, highlighting innovations in drug delivery, exercise interventions, artificial intelligence and computational biology, cancer stem cell plasticity, and translational research approaches. The meeting emphasised mechanistically informed strategies, multi-modal therapies, and the integration of patient-relevant models to improve therapeutic outcomes. The sessions collectively underscored the importance of combining biological, chemical, and physical approaches, as well as emerging tools in precision oncology, to address therapeutic resistance and enhance patient care.

## Simple Summary

The 2025 Irish Association for Cancer Research (IACR) annual meeting in Belfast highlighted advances across cancer prevention, therapy, and computational research. Key sessions explored the role of lifestyle interventions, including exercise, in supporting patient outcomes and potentially enhancing treatment efficacy. Cutting-edge drug delivery strategies were presented, including thermally and sonodynamically activated nanoparticles, designed to improve tumour-specific delivery while modulating the tumour microenvironment. Presentations on cancer stem cells and plasticity emphasised how adaptive tumour states drive therapy resistance, while computational approaches and AI were shown to uncover genetic vulnerabilities and optimize diagnostic imaging. Collectively, the meeting underscored the importance of integrative strategies combining lifestyle, mechanistic therapeutics, and data-driven approaches to improve patient care and inform future cancer therapies.

## 1. Introduction

The Irish Association for Cancer Research (IACR) is Ireland’s primary scientific society dedicated to advancing cancer research, fostering collaboration between basic scientists, translational researchers, and clinicians across the island of Ireland and internationally. The IACR annual conference serves as a key forum for disseminating emerging research and shaping national cancer research priorities. The 2025 annual meeting brought together over 300 delegates from academia, clinical medicine, and industry across multiple oral sessions and a dedicated poster programme. A notable feature of the IACR annual conference is the strong and meaningful contribution of IACR Patient and Public Involvement (PPI) members, which was embedded throughout the conference programme. A dedicated PPI strand provided a structured forum for patient advocates to engage directly with the research community, while patients and members of the public also participated in interactive workshop activities and played an active role in conference organisation, including serving as judges for poster and oral presentations in the early-career researchers competitions. In her presidential welcome, Prof. Jacintha O’Sullivan emphasised the breadth of the programme, which showcased cancer research spanning prevention, therapy, and translational science. The conference provided dedicated opportunities for early-career researchers to present their work, and awards were conferred for outstanding contributions to cancer research, culminating with the IACR Award presented to Prof. Tracy Robson (RCSI). This conference report highlights selected presentations chosen to represent the major thematic sessions of the 2025 meeting, as detailed in Table 1.

## 2. Exercise as an Adjunctive Cancer Therapy

It has been well established that exercise can influence cancer prevention and function as a supportive care intervention; however, the evidence for exercise as an effective treatment remains unclear. Professor Kerry Courneya’s (University of Calgary, Canada) research focuses on the role of exercise in cancer therapy. Non-interventional epidemiological studies have shown that leisure-time physical activity is linked to a lower risk of developing many cancer types [1]. Furthermore, exercise improves patient quality of life before, during, and after treatment, with several countries providing exercise recommendations during cancer, such as the American Society for Clinical Oncology (ASCO) Exercise, Diet, and Weight Management During Cancer Treatment guideline [2].

In the Irish Cancer Society Guest Lecture “The emerging role of exercise as a cancer treatment,” Prof. Courneya explained the challenges in understanding the optimal placement of exercise within the complex landscape of cancer therapy. He proposed that the positioning of exercise within different treatment combinations, sequences, and disease stages may affect efficacy and tolerance, forming the basis of the Exercise Across the Postdiagnosis Cancer Continuum (EPiCC) framework [3].

In early-stage cancer, preclinical studies simulating deferred treatment or active surveillance have tested exercise as a primary monotherapy with modest results. Prof. Courneya’s research in a cohort of prostate cancer patients demonstrated that 12 weeks of high-intensity interval training decreased markers of biochemical progression compared to usual care and improved cardiovascular fitness [4].

As cancer progresses, the influence of exercise on treatment efficacy becomes more complex. Preclinical rodent studies show benefits of concurrent exercise in enhancing therapy response [5]. Clinical studies in rectal and breast cancer indicate that exercise during active treatment can improve pathological complete response [6,7]. However, much of the current evidence derives from observational studies, which often avoid clinically relevant time points, fail to account for treatment type, and may miss early therapeutic responses.

Prof. Courneya’s EPiCC framework proposes the concept of precision exercise for cancer treatment, wherein efficacy depends on the right intervention, patient, and timing. Despite promising data, there remains a need for preclinical studies examining different exercise positions within various treatment combinations and for translational studies addressing diverse clinical oncology scenarios.

Emerging evidence suggests that systemic factors such as inflammation, metabolic health, and physical activity may also influence tumour cell plasticity and microenvironmental signalling, raising the possibility that lifestyle interventions could indirectly modulate adaptive therapeutic resistance. The mechanistic basis for these observations is an active area of investigation. Proposed pathways discussed at the session include reductions in circulating inflammatory cytokines, enhanced natural killer cell cytotoxicity, improved insulin sensitivity and consequent reductions in insulin-like growth factor signalling, and exercise-induced increases in tumour perfusion that may improve drug delivery. Understanding which biological pathways are activated—and under what exercise conditions—was highlighted as central to optimising exercise as an active treatment component rather than purely supportive care.

## 3. Advances in Drug Delivery for the Treatment of Cancer

Effective drug delivery to solid tumors remains one of the central challenges in oncology. Physical barriers within the tumor microenvironment—including dense stromal architecture, aberrant vasculature, elevated interstitial pressure, and immunosuppressive cell populations—impair the penetration and retention of conventional chemotherapeutics and limit the efficacy of emerging targeted agents. Addressing these barriers requires mechanistically informed delivery strategies that integrate chemical, physical, and biological approaches to achieve selective tumor accumulation while minimising systemic toxicity. The session on advances in drug delivery highlighted innovative approaches to overcoming these barriers.

Professor Robert Straubinger (Roswell Park, USA) discussed pancreatic ductal adenocarcinoma (PDAC), the fourth leading cause of cancer death, where conventional chemotherapy provides minimal survival benefit due to dense stromal barriers, poor vascular perfusion, and intrinsic or acquired drug resistance [8]. His laboratory focuses on mechanistically designed nanoparticle formulations and stromal normalisation strategies to enhance tumour-specific delivery. Using low-passage patient-derived xenograft models that recapitulate patient tumour architecture and delivery barriers, Straubinger demonstrated that transient inhibition of sonic Hedgehog signalling (sHHi) can reduce stromal fibrosis, restore vascular patency, and enhance intratumoral deposition of nanoparticle therapeutics. Stromal modulation also induces adaptive tumour–stromal responses, including activation of FGFR signalling and epithelial–mesenchymal transition programs. Incorporating FGFR inhibitors into a dual-hit treatment strategy counteracts these compensatory mechanisms, leading to improved tumour control and prolonged progression-free intervals in PDAC models [9,10,11,12,13,14].

Professor Wafa’ T. Al-Jamal (Queen’s University Belfast, UK) extended these concepts with thermally responsive liposomal platforms for solid tumours. She discussed low-temperature-sensitive liposomes (LTSL) and thermo-triggered liposomes to enhance local drug concentrations through on-demand release, demonstrating improved tumour control and survival in preclinical models [15,16]. Her group also investigated prostate-specific antigen–cleavable LTSL systems, which prolong circulation, enhance tumour selectivity, increase intratumoral drug accumulation, and reduce dose-limiting toxicity [17,18]. Beyond chemotherapy, magnetoliposome platforms capable of photothermal activation and MRI-guided delivery, when combined with immune checkpoint blockade, produced enhanced antitumour immune responses and increased infiltration of cytotoxic CD8+ T cells [19,20]. Next-generation photothermal agents, including indocyanine green J-aggregate-loaded liposomes, achieved efficient tumour targeting, favourable pharmacokinetics, and robust cytotoxicity in 2D and 3D tumour models [21,22,23].

Professor Anthony McHale (Ulster University, UK) presented nanoparticle-augmented sonodynamic therapy (SDT) in PDAC. Using pH-responsive polymer-coated CaO_2_ nanoparticles, selective activation within the acidic tumour microenvironment induced direct cytotoxicity and stromal modulation. SDT treatment increased the ratio of CD8+ cytotoxic T cells to regulatory T cells, eliciting an abscopal effect and demonstrating potential for metastatic disease models [24,25]. Stromal analyses revealed a shift toward a benign cancer-associated fibroblast phenotype, with increased Meflin and reduced αSMA and FAP expression, supporting a broader role for SDT in remodelling the tumour microenvironment to enhance therapeutic responsiveness.

Collectively, these presentations highlighted the integration of physical, chemical, and biological strategies to overcome intrinsic barriers of solid tumours, emphasising mechanistic design, microenvironmental modulation, and on-demand cytotoxic activation for improved chemotherapy and immunotherapy outcomes. By modulating stromal architecture and immune composition, these approaches may also influence tumour cell state transitions, highlighting a potential interface between microenvironment-targeted therapies and plasticity-driven resistance mechanisms. Speakers acknowledged that translating these platforms into the clinic presents significant challenges. Biodistribution and selective tumour accumulation without off-target toxicity remain key concerns. Manufacturing scalability for liposomal and polymer-coated nanoparticles, and regulatory pathways for combination physical-chemical-biological modalities, will need to be addressed as these approaches move toward clinical development. Patient-derived preclinical models and rigorous pharmacokinetic characterisation were highlighted as critical steps in building the translational evidence base for these systems.

## 4. Artificial Intelligence and Computational Oncology

The session on artificial intelligence (AI) and computational oncology highlighted computational methods applied to cancer genetics, tumour evolution, and imaging. Specifically, three distinct applications were presented: functional genomic dependency mapping using CRISPR–Cas9 screens to prioritise therapeutic targets; machine learning frameworks for identifying driver alterations at the individual tumour level; and AI-assisted clinical imaging for breast cancer screening and risk stratification. Presenters illustrated how analytical frameworks and machine learning tools are being used to support the interpretation of complex biological and clinical data.

Dr Francesco Iorio (Human Technopole, Italy) discussed large-scale functional genomic screening using CRISPR–Cas9 to map genetic dependencies across cancer cell lines and thereby prioritise potential therapeutic targets. In the context of broad efforts to identify vulnerabilities in defined tumour subtypes, previous studies have reported that the WRN helicase is a synthetic lethal dependency in cancers with microsatellite instability. To support the analysis of CRISPR screening data, Dr Iorio introduced CRISPRcleanR and its web application, a tool designed to facilitate processing, correction, and visualisation of pooled CRISPR–Cas9 screen results. The platform aims to improve robustness in downstream analysis and interpretation by providing accessible workflows for data handling and quality control [26].

Dr Francesca Ciccarelli (Barts Cancer Institute, London, UK) presented machine learning approaches for identifying patient-specific cancer driver genes, including the sysSVM framework [27]. By integrating mutational and systems-level features, this method aims to prioritise likely driver alterations at the individual tumour level. Using Barrett’s oesophagus as a model of early tumour evolution, her work illustrated how chronic inflammatory processes shape mutational landscapes and how individual genetic alterations, including CDKN2A loss, must be interpreted within a broader molecular context rather than as isolated determinants of progression. These findings emphasise the complexity of early carcinogenesis and the value of computational approaches in dissecting multi-step tumour evolution.

Dr Fredrik Strand (Karolinska Institutet, Sweeden) presented ongoing clinical research evaluating artificial intelligence in breast cancer screening and risk stratification. His work includes the prospective ScreenTrustCAD and ScreenTrustMRI trials, which assess AI-assisted mammography and AI-guided selection for supplemental MRI in population-based screening settings. The ScreenTrustCAD trial demonstrated that AI-supported mammography reading in a paired-reader, non-inferiority design maintained cancer detection performance while reducing radiologist workload [27]. The randomized ScreenTrustMRI study further explored AI-based risk assessment to identify women who may benefit from supplemental MRI, illustrating how machine learning models can support personalised screening strategies [28]. Strand also highlighted efforts to develop and externally validate multimodal AI models using curated, high-quality imaging datasets linked to clinical outcomes, including multi-institutional validation studies of mammography-based risk prediction models [29] and reinforcement learning approaches to optimise screening policies [30]. These studies reflect a broader strategy of integrating AI tools with radiologist expertise, supported by multicentre validation platforms such as the Swedish VAI-B initiative. Collectively, this work illustrates how rigorously evaluated AI systems may contribute to risk-adapted screening, workflow optimisation, and evidence-based implementation in breast imaging.

Together, the presentations underscored the expanding role of computational and AI-based methods in cancer research. From functional genomics to driver gene identification and imaging support, these approaches contribute analytical frameworks that complement experimental and clinical studies, enabling systematic interpretation of large and multi-dimensional data relevant to precision oncology. Notably, computational identification of tumour-specific vulnerabilities and driver alterations complements mechanistically designed drug delivery platforms, enabling more precise matching of targeted therapeutics to defined molecular subgroups.

## 5. Emerging Hallmarks of Cancer Stem Cells and Plasticity

Cancer stem cells and cellular plasticity have emerged as defining features of tumour evolution, therapeutic resistance, and metastatic progression. Rather than being governed by a fixed hierarchical model, tumour cell populations exhibit remarkable phenotypic flexibility, transitioning between stem-like and differentiated states in response to microenvironmental and therapeutic pressures [31,32]. Such state transitions are orchestrated by complex networks of transcriptional, epigenetic, and signalling regulators and are now recognised as critical determinants of tumour persistence and relapse. This session, “Emerging Hallmarks of Cancer Stem Cells and Plasticity,” showcased recent mechanistic and translational advances revealing how cellular plasticity drives tumour evolution, metastatic progression, and therapeutic resistance. Presentations by Prof Christine Chaffer, Dr. Anna Golebiewska, and Dr. Kevin Myant highlighted diverse mechanisms through which tumour cells acquire adaptive stem-like and lineage-plastic states in response to therapy and microenvironmental pressures. Collectively, these studies illustrated how targeting the regulatory networks governing cellular plasticity—either pharmacologically, epigenetically, or through modulation of the tumour microenvironment—may open new avenues for more durable therapeutic strategies.

Prof Christine Chaffer’s (Garvan Institute of Medical Research, Australia) work explores the mechanisms by which chemotherapy induces cell-state plasticity in aggressive breast cancers, and how this can be therapeutically intercepted. Her lab has demonstrated that triple-negative breast cancer cells possess the capacity to switch reversibly between epithelial and mesenchymal-like states, a process mediated by the transcription factor ZEB1 and associated epithelial–mesenchymal transition (EMT) programs [33,34]. This intrinsic plasticity allows non-stem cancer cells to acquire stem-like features following cytotoxic stress, fuelling minimal residual disease and recurrence [35,36]. Recent work from the Chaffer group has identified androgen receptor (AR) signalling as a key driver of chemotherapy-induced reprogramming in triple-negative breast cancer. Functional studies revealed that AR activation promotes EMT and cancer stem cells function, while AR inhibition suppresses both [37]. The group is developing seviteronel, an inhibitor of AR, as a state-gating therapeutic designed to prevent cell-state transitions triggered by chemotherapy. In preclinical triple-negative breast cancer models, seviteronel combined with taxane chemotherapy effectively blocked the induction of stem-like states and reduced metastasis. Mechanistically, seviteronel transcriptionally mimics AR knockdown (siAR), repressing EMT-related pathways and PI3K signalling. Early clinical trial data (4CAST study, Phase Ib) indicate that seviteronel is safe in combination with taxanes and yields promising responses [38]. These findings suggest a shift in therapeutic strategy, moving beyond targeting fixed cancer cell populations toward limiting the development of therapy-resistant states.

Dr. Anna Golebiewska (Luxembourg Institute of Health, Luxembourg) presented a comprehensive systems-level analysis of glioblastoma as a model of extreme tumour cell plasticity. Her work from the NORLUX Neuro-Oncology Laboratory shows that glioblastoma cells exist in a spectrum of transcriptional and phenotypic states that continuously interconvert, rather than following a unidirectional, hierarchical stem cell model [39]. Using patient-derived models and single-cell transcriptomics, her team developed a non-hierarchical, stochastic model of cell-state transitions, demonstrating that each subpopulation re-establishes tumour heterogeneity over time [40]. This dynamic equilibrium is sustained through co-evolution between tumour cells and the microenvironment, where environmental stressors such as hypoxia, therapy, and immune infiltration select for specific adaptive states. Dr. Golebiewska emphasized the role of the immune microenvironment as a key modulator of plasticity. Her group’s recent work illustrates how glioblastoma cells “educate” immune components, including microglia, to adopt heterogeneous phenotypic states with phagocytic and dendritic cell-like features in patient tumours and patient-derived orthotopic xenografts [41]. Advanced spatial and computational tools have been used to reconstruct intercellular signalling networks, revealing how therapy-induced changes in the tumour ecosystem foster adaptive resistance. Together, these findings portray glioblastoma as a highly plastic and co-evolving ecosystem in which tumour and immune cells dynamically reshape one another. 

Dr. Kevin Myant presented a talk entitled “Loss of Colonic Lineage Fidelity Permits Induction of Squamous-Like Plasticity and Metastasis in Colorectal Cancer” highlighting recent work, subsequently published in Nature, demonstrating how disruption of epithelial lineage identity drives tumour progression through cellular plasticity [42]. Using genetically engineered and patient-derived organoid models together with chromatin accessibility and transcriptomic analyses, the study showed that loss of the chromatin remodeller ATRX destabilises colonic epithelial differentiation programs and promotes the emergence of highly plastic mesenchymal and squamous-like cellular states. These altered states were associated with enhanced invasiveness, metastatic dissemination, and poor clinical outcome. Mechanistically, ATRX loss impaired lineage-specifying transcriptional networks, including HNF4A-dependent colonic lineage programs, enabling widespread epigenetic reprogramming and adaptive cell-state transitions independent of additional genetic alterations. Collectively, these findings position loss of lineage fidelity as a critical mechanism underlying metastatic evolution and reinforce the broader concept emerging throughout the session that tumour plasticity represents a central driver of therapeutic resistance and disease progression [42].

The presentations by Chaffer, Golebiewska, and Myant collectively underscored cellular plasticity as a defining hallmark of tumour evolution, metastatic progression, and therapeutic resistance. Across breast cancer, glioblastoma, and colorectal cancer, tumour cells were shown to dynamically transition between phenotypic states in response to intrinsic transcriptional programs, epigenetic reprogramming, microenvironmental pressures, and therapy-induced stress. Whether driven by AR-mediated state switching in triple-negative breast cancer, tumour–immune co-evolution in glioblastoma, or loss of lineage fidelity in colorectal cancer, these studies highlighted the remarkable adaptive capacity of malignant cells. Together, the work presented in this session supports a growing paradigm shift in cancer biology: durable therapeutic responses may depend not only on eliminating tumour cells, but on disrupting the regulatory networks that enable cellular plasticity and adaptive state transitions.

## 6. Nutrition, Obesity, and Host Factors in Cancer Progression and Treatment Response

There is growing recognition that cancer outcomes are shaped not only by tumour biology but also by the systemic physiological context of the patient. Malnutrition, sarcopenia, metabolic dysfunction, hormonal imbalances, and environmental exposures are increasingly understood as modulators of cancer risk, disease progression, and treatment tolerance. This session explored the critical influence of host factors, including malnutrition, sarcopenia, metabolic health, and environmental exposures, on cancer progression, treatment tolerance, and patient outcomes.

Dr. Oonagh Griffin (St. Vincent’s University Hospital, Ireland) outlined the profound impact of malnutrition and muscle wasting in pancreatic cancer, where unintentional weight loss affects up to 80% of patients and significantly reduces survival even in resectable disease. Muscle depletion is an adverse prognostic feature in metastatic pancreatic cancer [43], while preservation of muscle indices during neoadjuvant chemotherapy correlates with improved treatment outcomes [44]. Malnutrition arises from multiple factors: inadequate intake due to pain, anorexia, and food aversion; impaired nutrient utilisation caused by tumour-driven metabolic reprogramming and systemic inflammation; and malabsorption secondary to exocrine insufficiency or surgical resections [45,46,47]. Tumour-induced cachexia drives accelerated protein turnover, lipolysis, and insulin resistance, compounding skeletal muscle loss [48].

Dr. Griffin highlighted the FEED study, a 12-week dietitian-led multimodal intervention integrating dietary counselling, pancreatic enzyme replacement therapy, exercise, and fish oil supplementation [49]. Conducted alongside neoadjuvant chemotherapy, the study demonstrated feasibility [49]. Building on these findings, the ongoing FEED Trial (NCT06149546) has added a physiotherapist-supervised exercise component and expanded recruitment to validate clinical efficacy and integrate early nutrition support into routine PDAC care [50].

Dr. Gwen Murphy (Imperial College London, UK) addressed trends in gastric cancer, highlighting the rise in early-onset disease among individuals aged 25–39, now representing over 30% of US cases [51]. Analysis of registries including Northern Ireland Cancer Registry (1993–2021) showed a persistently high proportion of stage IV diagnoses and poor overall survival [52]. The lecture highlighted under-identification of premalignant lesions remains a significant challenge [53]. Dr. Murphy presented evidence from cohort studies linking hormonal and metabolic biomarkers to gastric cancer risk. Low circulating ghrelin was associated with increased risk of gastric non-cardia and oesophagogastric junction adenocarcinoma [54,55], while elevated gastrin levels [56] and low vitamin B12 [57], often a consequence of autoimmune gastritis, correlated with heightened risk. She highlighted an ongoing study (The Gastric Hormone Biomarkers of Preneoplastic Lesions (GEM) study), using a “gastric super-panel,” combining gastrin, ghrelin, vitamin B12 and pepsinogen, to improve early detection and triage in low-incidence populations, emphasising the potential of leveraging gastrointestinal physiology alongside high-throughput biomarker platforms.

Professor Lisa Connolly (Queen’s University Belfast, UK) focused on the pervasive effects of endocrine-disrupting chemicals (EDCs) from environmental, dietary, and packaging sources. Exposure to EDCs has been linked to obesity, diabetes, reproductive dysfunction, and hormone-related cancers [58,59]. She highlighted in vitro work showing that steviol, a metabolite of the natural sweetener stevia, antagonises progesterone receptor signalling and alters steroidogenesis, increasing progesterone production at high concentrations [60].

Professor Connolly also addressed the development of safer and sustainable bio-based food packaging where she described bioassay-guided development of novel polymers, demonstrating markedly reduced estrogenic and anti-androgenic activity compared with conventional petroleum-based plastics [61]. Such approaches could minimise human exposure to hormonally active leachates while reducing reliance on non-renewable fossil fuels, highlighting the intersection of environmental chemistry and cancer risk mitigation.

This session emphasised the importance of host factors in modulating cancer outcomes. Malnutrition, sarcopenia, and metabolic or environmental exposures can limit treatment tolerance and worsen prognosis. Multimodal interventions, early nutritional assessment, biomarker-informed risk stratification, and safer environmental practices represent actionable strategies to optimize patient care and improve long-term survival.

## 7. IACR Award for Outstanding Contribution to Cancer Medicine and Research

The conference concluded with the presentation of the IACR Award for Outstanding Contribution to Cancer Medicine and Research, recognizing sustained excellence in translational cancer research. The IACR Award for Outstanding Contribution to Cancer Medicine and Research is conferred annually to recognise researchers whose work has made a sustained and significant impact on cancer biology or clinical oncology. The 2025 award lecture illustrated the translational arc from fundamental molecular discovery through to clinical trial. The Award was presented to Professor Tracy Robson, Deputy Vice Chancellor for Academic Affairs at the Royal College of Surgeons in Ireland, in recognition of her sustained and influential contributions to translational cancer biology. Her award lecture, entitled “My Journey of Discovery from Cancer to Obesity: A Tale of One Protein with Many Functions,” traced a scientific journey defined by curiosity, resilience, and the evolving complexity of biological discovery.

Professor Robson outlined her early training in molecular cancer cell biology, and her journey to PhD completion at Imperial College London, where she investigated mammalian genes induced by low-dose ionizing radiation and their relevance to cancer induction and therapy [62,63,64]. This work was further developed during her time with the Radiation Science Group at the University of Ulster, where her research laid the foundation for a long-standing interest in stress response pathways and tumour adaptation. Central to this journey was the discovery and characterisation of FK506-binding protein-like (FKBPL), a divergent member of the immunophilin family distinguished by a non-functional peptidyl-prolyl isomerase domain and functional tetratricopeptide repeat motifs.

Professor Robson described how FKBPL emerged as a multifaceted regulator of cancer biology, with context-dependent roles spanning hormone responsiveness, angiogenesis, stemness, and therapeutic resistance. Increased FKBPL expression was shown to sensitise breast cancer cells to oestrogen deprivation and predict responsiveness to endocrine therapy [65,66]. Large-scale meta-analyses subsequently established FKBPL as an independent prognostic biomarker for breast cancer–specific and progression-free survival [66,67]. 

Beyond biomarker discovery, Professor Robson highlighted the translational development of FKBPL-derived therapeutic peptides, including AD-01 and the clinical candidate ALM201 [5]. These agents demonstrated potent anti-angiogenic and anti–cancer stem cell activity via CD44-dependent mechanisms and showed efficacy across multiple tumour types, both as monotherapies and in combination with chemotherapy [68,69]. A first-in-human phase I trial confirmed ALM201 to be safe and well tolerated, leading to orphan drug designation in ovarian cancer [70].

Professor Robson highlighted her involvement within Precision Oncology Ireland, where the FKBPL programme targets the significant unmet clinical need in high-grade serous ovarian cancer, a disease characterised by high relapse rates and poor survival. To overcome limitations of peptide-based therapies, her group is developing a FKBPL-based nanomedicine using delivery of the full-length FKBPL gene via the tumour-selective RALA nanoparticle platform [71]. RALA-FKBPL enables sustained, tumour-specific expression and has demonstrated enhanced efficacy in preclinical models. In parallel, working with Professor Donal Brennan, RNA sequencing of primary tumours is being used to develop a companion gene expression signature to identify patients most likely to benefit from FKBPL-based therapy [71].

In an extension beyond oncology, Professor Robson highlighted emerging work within her group investigating the role of FKBPL in metabolically unhealthy obesity, using both preclinical models and human cohorts. Early findings have prompted investigation of FKBPL gene delivery approaches to modulate metabolic dysfunction, leading to the broader question of whether FKBPL may protect against obesity-associated cancer development. This question is particularly relevant in the context of Barrett’s oesophagus and oesophageal adenocarcinoma, as well as hepatocellular carcinoma, where obesity and metabolic dysfunction are major risk factors and survival remains poor. Together, these studies highlight the potential intersection of FKBPL with metabolic disease and cancer risk, opening new avenues for prevention-focused and cross-disease research.

Professor Robson concluded by emphasising that FKBPL is both powerful and complex, that tumour and tissue context is critical, and that scientific discovery rarely follows a linear path. Her closing message—science never goes in straight lines; never give up—resonated as both a personal reflection and a guiding principle for translational and precision oncology research.

This lecture encapsulated the conference’s broader emphasis on mechanistic insight, translational innovation, and the expanding interface between cancer biology, metabolism, and prevention.

## 8. Discussion

The IACR Annual Conference highlighted the increasingly interconnected nature of contemporary cancer research, emphasising that durable therapeutic progress requires integration across molecular biology, host physiology, technological innovation, and clinical translation. Across sessions, a recurring theme was the need to address not only tumour-intrinsic drivers but also the dynamic and adaptive processes that shape treatment response.

Tumour plasticity and adaptive resistance emerged as a central conceptual thread. Presentations on cancer stem cells and cellular state transitions underscored that malignant cells are not static entities but exist within flexible phenotypic landscapes influenced by therapeutic pressure and microenvironmental cues [31,32]. Importantly, several sessions illustrated complementary strategies to intercept these adaptive processes. Microenvironment-targeted drug delivery platforms, including stromal modulation and thermally or sonodynamically activated nanoparticles [15,16,24,25], aim to overcome structural and immune barriers that contribute to therapeutic resistance. By reshaping tumour architecture and immune composition, these approaches may indirectly influence the cellular state transitions that underpin recurrence and metastasis [33,34,39,42].

At the same time, advances in computational oncology provide systematic tools to identify tumour-specific vulnerabilities and evolutionary trajectories. Large-scale CRISPR–Cas9 dependency mapping [26] and patient-level driver identification frameworks [27] offer mechanistic insight into genetic dependencies that may be exploited therapeutically. In parallel, AI-driven imaging and risk modelling approaches [27,28,29,30] demonstrate how computational methods can be rigorously evaluated in clinical screening programmes, supporting workflow optimisation and risk-adapted strategies. Together, these technologies enable increasingly precise alignment between molecular vulnerabilities, therapeutic targeting, and clinical decision-making.

A second major theme was the influence of host factors on cancer progression and treatment response. Sessions addressing exercise, nutrition, sarcopenia, metabolic health, and environmental exposures emphasised that tumour biology cannot be disentangled from systemic physiology. Systemic inflammation, metabolic dysregulation, and endocrine signalling may in turn influence tumour cell state transitions, reinforcing the interconnectedness of host biology and adaptive resistance mechanisms. Interventions such as structured exercise programmes [3,5,6,7] and multimodal nutritional support [49,50] were presented not merely as supportive care, but as potential modulators of treatment tolerance and therapeutic efficacy. These perspectives reinforce a broader shift toward patient-centred oncology, in which systemic inflammation, metabolic status, and environmental exposures are recognised as contributors to therapeutic outcomes [43,44,48,58,59].

Importantly, these domains are not independent. Computational tools can inform personalised screening and therapeutic stratification; drug delivery platforms can remodel the tumour microenvironment; lifestyle and metabolic interventions may influence inflammatory and signalling pathways that shape tumour evolution. Across prevention, treatment, and translational research, speakers consistently highlighted the necessity of multimodal and adaptive strategies that acknowledge biological complexity and tumour–host interaction.

Finally, the translational trajectory of FKBPL, from molecular discovery to biomarker development [65,66,67], peptide therapeutics [68,69], nanomedicine [71], and emerging metabolic applications, exemplifies the non-linear but integrative nature of cancer research. This pathway reflects a broader conference message: progress in oncology increasingly depends on the convergence of mechanistic insight, technological innovation, and clinical implementation. As a conference report, this article reflects the scope and emphasis of the 2025 IACR meeting as presented and does not aim to provide a comprehensive or systematic appraisal of each field. Many of the approaches discussed remain at preclinical or early clinical stages, and translational barriers—including patient selection, scalability, long-term safety, and the complexity of multi-modal combination strategies—will require sustained investigation before their full clinical potential is realised. The IACR meeting provided an important forum for identifying where convergence across these domains is already beginning to generate meaningful progress.

## 9. Conclusions

The 2025 IACR Annual Conference showcased the breadth of contemporary cancer research, spanning molecular mechanisms, technological innovation, and patient-focused interventions. Across sessions, the meeting demonstrated the value of integrating biological, clinical, and computational perspectives to address the complexity of cancer as a disease.

The conference highlighted significant advances in mechanistically guided drug delivery, computational oncology, and exercise and nutritional science, alongside a growing understanding of cancer stem cell plasticity and host factors. Computational and AI-based approaches enabled more precise identification of cancer drivers and therapeutic vulnerabilities, while AI-assisted imaging supported risk-adapted clinical screening. Targeting cell-state transitions and the regulatory networks governing plasticity was presented as a priority avenue to reduce resistance and improve long-term outcomes. Overall, the meeting reinforced that effective cancer prevention and treatment depend on integrated, patient-centred strategies that consider both tumour biology and host factors.

## Figures and Tables

**Table 1:** Summary of 2025 IACR Annual Conference sessions, plenary speakers, and clinical implications.

Session	Plenary Speaker(s)	Clinical Implications
Exercise as an Adjunctive Cancer Therapy	Prof. Kerry Courneya (University of Calgary, Canada)	Exercise may function as an active treatment adjunct beyond supportive care. Optimal timing, type, and intensity relative to treatment requires prospective evaluation. Mechanistic studies on immune modulation and tumour perfusion are needed to guide clinical integration.
Advances in Drug Delivery for the Treatment of Cancer	Prof. Robert Straubinger (Roswell Park, USA); Prof. Wafa’ T. Al-Jamal (Queen’s University Belfast, UK); Prof. Anthony McHale (Ulster University, UK)	Mechanistically designed delivery platforms can overcome stromal and vascular barriers in solid tumours. Biodistribution, scalability, and regulatory pathways for combination modalities remain key translational challenges requiring patient-derived model validation.
Artificial Intelligence and Computational Oncology	Dr. Francesco Iorio (Human Technopole, Italy); Dr. Francesca Ciccarelli (Barts Cancer Institute, UK); Dr. Fredrik Strand (Karolinska Institutet, Sweden)	AI tools can augment genomic target identification, patient-specific driver gene prioritisation, and clinical imaging workflows. Prospective multicentre validation is essential before widespread clinical implementation.
Emerging Hallmarks of Cancer Stem Cells and Plasticity	Prof. Christine Chaffer (Garvan Institute, Australia); Dr. Anna Golebiewska (Luxembourg Institute of Health); Dr. Kevin Myant (University of Edinburgh, UK)	Targeting cell-state transitions and the regulatory networks governing plasticity may complement cytotoxic strategies and reduce therapeutic resistance. Epigenetic and microenvironmental modulators represent actionable therapeutic avenues.
Nutrition, Obesity, and Host Factors in Cancer	Dr. Oonagh Griffin (St. Vincent’s University Hospital, Ireland); Dr. Gwen Murphy (Imperial College London, UK); Prof. Lisa Connolly (Queen’s University Belfast, UK)	Host metabolic and nutritional status should be integrated routinely into cancer management. Biomarker panels may enable earlier risk stratification. Reducing environmental endocrine-disrupting chemical exposure represents a prevention-focused intervention.
IACR Award Lecture	Prof. Tracy Robson (RCSI, Ireland)	FKBPL exemplifies the translational journey from molecular discovery to clinical trial. Nanomedicine and companion diagnostic strategies are applicable across tumour types. The intersection of FKBPL with metabolic disease opens prevention-focused research opportunities.

## Data Availability

Not applicable.

## Abbreviations

The following abbreviations are used in this manuscript:

AI	Artificial Intelligence
AR	Androgen receptor
CAF	Cancer-associated fibroblast
CRISPR	Clustered regularly interspaced short palindromic repeats
EMT	Epithelial–mesenchymal transition
EPiCC	Exercise Across the Postdiagnosis Cancer Continuum
FGFR	Fibroblast growth factor receptor
FKBPL	FK506-binding protein-like
HGSOC	High-grade serous ovarian cancer
LTSL	Low-temperature-sensitive liposomes
MRI	Magnetic resonance imaging
PDAC	Pancreatic ductal adenocarcinoma
PDX	Patient-derived xenograft
SDT	Sonodynamic therapy
TSL	Temperature-sensitive liposomes
